# Safety and feasibility of hemodynamic pulmonary artery pressure monitoring using the CardioMEMS device in LVAD management

**DOI:** 10.1111/jocs.15767

**Published:** 2021-06-22

**Authors:** Jesse F. Veenis, Sumant P. Radhoe, Nicolas M. van Mieghem, Olivier C. Manintveld, Jos A. Bekkers, Kadir Caliskan, Ad J.J.C. Bogers, Felix Zijlstra, Jasper J. Brugts

**Affiliations:** ^1^ Department of Cardiology, Erasmus MC, Thorax Center University Medical Center Rotterdam Rotterdam The Netherlands; ^2^ Department of Interventional Cardiology, Erasmus MC, Thorax Center University Medical Center Rotterdam Rotterdam The Netherlands; ^3^ Department of Cardiothoracic Surgery, Erasmus MC, Thorax Center University Medical Center Rotterdam Rotterdam The Netherlands

**Keywords:** CardioMEMS, heart failure, HeartMate 3, LVAD, remote monitoring

## Abstract

**Background:**

There is a clinical need for additional remote tools to improve left ventricular assist device (LVAD) patient management. The aim of this pilot concept study was to assess the safety and feasibility of optimizing patient management with add‐on remote hemodynamic monitoring using the CardioMEMS in LVAD patients during different treatment stages.

**Methods:**

Ten consecutive patients accepted and clinically ready for (semi‐) elective HeartMate 3 LVAD surgery were included. All patients received a CardioMEMS to optimize filling pressure before surgery. Patients were categorized into those with normal mean pulmonary artery pressure (mPAP) (≤25 mmHg, *n* = 4) or elevated mPAP (>25 mmHg, *n* = 6), and compared to a historical cohort (*n* = 20). Endpoints were CardioMEMS device safety and a combined endpoint of all‐cause mortality, acute kidney injury, renal replacement therapy and/or right ventricular failure at 1‐year follow‐up. Additionally, we investigated hospital‐free survival and improvement in quality of life (QoL) and exercise tolerance.

**Results:**

No safety issues or signal interferences were observed. The combined endpoint occurred in 60% of historical controls, 0% in normal and 83% in elevated mPAP group. Post‐discharge, the hospital‐free survival was significantly better, and the QoL improved more in the normal compared to the elevated mPAP group.

**Conclusion:**

Remote hemodynamic monitoring in LVAD patients is safe and feasible with the CardioMEMS, which could be used to identify patients at elevated risk of complications as well as optimize patient management remotely during the out‐patient phase with less frequent hospitalizations. Larger pivotal studies are warranted to test the hypothesis generated from this concept study.

## 1 INTRODUCTION

Left ventricular assists device (LVAD) therapy is a rapidly growing treatment option for end‐stage heart failure (HF) patients refractory to medical treatment.^1^ Due to technological improvements and increasing experience with LVAD therapy, the overall survival of LVAD patients has significantly improved since the early 2000s.^2^ Despite these improvements, early and late LVAD‐related complications, such as right ventricular (RV) failure, acute kidney injury (AKI), LVAD‐related infections and major gastro‐intestinal bleedings, are still frequent.^3^, ^4^, ^5^ Rehospitalization and complication rates in the out‐patient phase of LVAD patients remain very high.^6^ There is a great clinical need for physicians treating LVAD patients to have additional tools to monitor patient management, especially remotely outside the hospital. Currently, no remote monitoring tools are used in LVAD care, except physical signs, such as weight and rhythm.

LVAD management is mostly guided by signs and symptoms during the physical examination, and incidental monitoring of the static pump measurements provided by the LVAD during out‐patient visits.^7^ Recently, remote hemodynamic monitoring using the CardioMEMS device (Abbott Inc.) is safe and effective in chronic HF patients.^8^ A new concept is to use the CardioMEMS device as a hybrid with the LVAD device, which has not been performed worldwide in a prospective manner.

We hypothesized that the additional hemodynamic data provided by CardioMEMS could aid physicians involved in LVAD management in three stages of treatment:

Pre‐LVAD surgery, the hemodynamic feedback can be used to optimize patients towards surgery by decongesting the kidney and RV. Thereby, the risk of renal or RV failure can be reduced, or if elevated pulmonary artery pressure (PAP) remains one can take additional measures to timely support for the RV with higher inotropes or temporary assist devices for the RV and/or early start of dialysis.

At the intensive care unit (ICU), noninvasive hemodynamic feedback could aid in optimizing fluid state (potentially restrict the fluid overload during the first days post‐LVAD surgery, which strains the RV) as well as to optimize pump settings on top of echocardiography. While some can argue this phase could be managed by the regular Swan‐Ganz catheter, one is more at risk for complications, such as bleeding, pneumothorax, rhythm disturbances and infections.

CardioMEMS can also prove its potential after discharge from the ICU to the regular ward and out‐patient clinic to individualize patient care, including fluid state, and potentially reduce the number of hospitalizations, early discovery of LVAD‐related complications and improve quality of life.

Therefore, we set up a pilot concept study to test this hypothesis for assessing the safety and feasibility of using CardioMEMS in the preoperative period as well as the ICU and out‐patient phase in LVAD patients.^9^ If proven feasible and clinically useful, these findings will be very clinically relevant to further study in larger pivotal studies to improve LVAD patient management.

## 2 MATERIALS AND METHODS

A detailed description of the design and methods of the HEMO‐VAD pilot study has been published previously.^9^ In brief, ten consecutive chronic HF patients with New‐York Heart Association functional Class ≥III and Interagency Registry for Mechanically Assisted Circulatory Support (INTERMACS) Class 2–5, scheduled for (semi‐) elective LVAD implantation between November 2017 and March 2019 were enrolled. Patients with significant RV dysfunction were excluded from participation in this study, defined as a tricuspid annular plane systolic excursion less than 13 mm, visually impaired RV function, or dilation on echocardiogram. All patients received a HeartMate 3 (HM3; Abbott Inc.) device in our institution. For insight, we additionally present a cohort of all historical (semi‐) elective LVAD recipients, fulfilling the same requirements in this analysis in the period of March 2016–November 2017 with HM3 without remote care. The local medical ethics committee from the Erasmus Medical Center Rotterdam approved the study (27 July 2017, MEC nr. 2017‐342), all patients provided informed consent, and the study complies with the Declaration of Helsinki.

### 2.1 CardioMEMS cohort

The ten enrolled patients received a CardioMEMS device at baseline, within one day of enrollment at the heart team decision when the patient was accepted for LVAD and deemed clinically ready for surgery (normally would have proceeded towards surgery directly). During a right heart catheterization, the CardioMEMS device is implanted, using access via the femoral vein. Pulmonary arteriogram is used to identify an appropriate target vessel, based on the vessel size and location. When the target vessel is identified, the CardioMEMS delivery system is inserted over a guidewire, and advanced to the target location, where the CardioMEMS device is released. After implantation, a Swan Ganz catheter is used to calibrated the CardioMEMS system. All enrolled patients received their CardioMEMS device

The CardioMEMS device allowed for daily PAP monitoring in the perioperative as well as the postoperative period. Before LVAD surgery, the medical treatment was optimized using the hemodynamic feedback, on top of the standard care, with the goal to hemodynamically optimize the patients. The central aims were to reach euvolemia and to normalize the mean PAP (≤25 mmHg) by titrating the dose of diuretic and vasodilator drugs at the discretion of the treating physician. The timing of LVAD surgery was at the discretion of the physician and surgeon, and determined by either normalized PAP, the urgency of surgery, or not responding pressure trend to medical changes. The duration of this optimization period after the CardioMEMS implant was a minimum of 1 day or a maximum of 2 weeks.

Post‐LVAD surgery, the CardioMEMS device was used to monitor the hemodynamic status of the patients at the ICU, clinical ward, and out‐patient (at home). During this period, the central aim was to normalize and/or to maintain a normal mean PAP (mPAP) (≤25 mmHg), if possible, by optimizing HF treatment and pump settings.

### 2.2 Historical cohort

For reference, we added a historical cohort, being all consecutive patients with (semi‐) elective HM3 LVAD surgery between March 2016–November 2017 in Erasmus Medical Center following the same inclusion criteria as for the CardioMEMS cohort. All patients who were mechanically supported (with an intra‐aortic balloon pump or extracorporeal membrane oxygenation) ≤5 days before LVAD surgery (INTERMACS 1) were excluded.

### 2.3 Safety endpoints

The endpoints of the safety analysis were freedom of sensor failures at 1‐year, freedom of device‐related complications at 1‐year, and the freedom of signal malfunction or interference with the CardioMEMS device and HM3.

### 2.4 Clinical endpoints

The primary endpoint of this analysis was the outcome 1‐year post‐LVAD surgery, assessed as a composite of all‐cause mortality, AKI, and/or the need for renal replacement therapy (RRT), and/or RV‐failure. Secondary endpoints were all‐cause mortality, AKI and/or RRT, RV‐failure, as well as all‐cause hospitalization, changes of mPAP post‐LVAD surgery, the number of medication changes post‐LVAD surgery, and changes in quality of life (assessed using the EQ‐5D‐5L, Kansas City cardiomyopathy questionnaire and patient health questionnaire‐9 (PHQ‐9) questionnaires) and functional performance defined as 6‐min walking distance during 1‐year of out‐patient remote management of LVAD patients with CardioMEMS.

AKI was defined as a minimum 1.5 times increase of baseline serum creatinine during the first seven days post‐LVAD implantation, according to the kidney disease improving global outcome criteria.^10^ RV‐failure was defined as the need of continuous inotropic support for ≥14 days, (temporary) right ventricular assist device support or nitric oxide ventilation for ≥48 h.^11^

### 2.5 Statistical analysis

Continuous data are expressed as median and interquartile range and compared by the Mann–Whitney *U* test. Categorical data are expressed as counts and percentages and compared by the two‐sided Fisher's exact test. The probability of survival/combined endpoint was calculated using the Kaplan‐Meier method and compared using the log‐rank test (time‐to‐first event analysis). Changes in quality of life between baseline and 3, 6, 9, and 12 months of follow‐up were analyzed using the Wilcoxon sum ranked test. A two‐sided *p* value of 0.05 or lower was considered statistically significant.

The CardioMEMS cohort was stratified into two groups based on mPAP provided by the CardioMEMS: patients with normalized mPAP, defined as a pre‐LVAD surgery mPAP ≤ 25 mmHg and those with an elevated mPAP, defined as a pre‐LVAD surgery mPAP > 25 mmHg. In the survival analysis, hospitalization‐free survival analysis, and changes in quality of life, the historical cohort was compared to the CardioMEMS cohort.

All statistical analyses were performed using Statistical Package for Social Sciences, version 25.0 (SPSS Inc.).

## 3 RESULTS

In total, 30 patients were included in this analysis, ten patients in the CardioMEMS cohort, and 20 patients in the historical cohort. The baseline characteristics of these 30 patients are shown in Table 1. The median age was 60 (53–66) years, and 87% of patients were men. The median RA pressure was 5.0 [2.3‐9.8], and the median systolic, diastolic, and mean PAP were 35 (28–46), 17 (12–23), and 24 (21–32) mmHg, respectively, at baseline.

**Table 1:** Baseline characteristics

	**CardioMEMS cohort (*n* = 10)**				
	Overall population (*n* = 10)	**Elevated mPAP patients (*n* = 6)**	**Normalized mPAP patients (*n* = 4)**	***p* value**	**Historical cohort (*n* = 20)**
Age (years)	60.1 (52.4–63.0)	60.3 (51.6–66.3)	58.7 (53.4–61.9)	0.670	60.1 (53.9–65.8)
Male gender (%)	7 (70.0)	4 (66.7)	3 (75.0)	1.000	19 (95.0)
BMI (kg/m^2^)	27.2 (23.1–28.6)	27.2 (23.5–29.2)	24.8 (21.5–30.6)	0.670	24.6 (20.2–26.3)
Systolic BP (mmHg)	98.5 (89.0–108.5)	101.5 (88.8–115.3)	95.5 (89.3–102.5)	0.593	95.5 (90.3–100.0)
Diastolic BP (mmHg)	66.5 (57.0–72.8)	65.5 (50.3–81.3)	66.5 (60.8–70.0)	0.915	62.0 (60.0–68.0)
Heart rate (/min)	70.0 (67.5–78.0)	70.0 (64.3–84.0)	71.5 (68.5–75.3)	0.829	73.5 (68.0–80.8)
History					
Myocardial infarction	4 (40.0)	4 (66.7)	0 (0.0)	0.076	11 (55.0)
CABG	2 (20.0)	2 (33.3)	0 (0.0)	0.467	6 (30.0)
PCI	‐	‐	‐	‐	7 (35.0)
Atrial fibrillation	4 (40.0)	3 (50.0)	1 (25.0)	0.571	9 (45.0)
Diabetes mellitus	4 (40.0)	2 (33.3)	2 (50.0)	1.000	6 (30.0)
Renal insufficiency	8 (80.0)	5 (83.3)	3 (75.0)	1.000	12 (60.0)
TIA/CVA	2 (20.0)	1 (16.7)	1 (25.0)	1.000	2 (10.0)
Laboratory values					
Creatinine (µmol/L)	159.5 (124.5–191.0)	174.5 (156.5–206.0)	121.0 (109.5–152.0)	0.032	163.0 (136.5–209.3)
e‐GFR (ml/min)	38.5 (31.5–47.5)	33.5 (27.8–39.0)	50.0 (40.5–63.3)	0.019	40.5 (29.3–48.8)
NT‐proBNP (pmol/L)	476.5 (297.8–565.0)	531.5 (328.8–655.0)	372.0 (263.3–554.5)	0.522	679.0 (399.0–1677.0)
Bilirubin (µmol/L)	13.0 (10.5–20.3)	11.0 (8.3–20.3)	15.0 (12.5–19.8)	0.238	17.5 (14.5–25.8)
Echocardiogram					
Left ventricular ejection fraction (%)	19.0 (13.0–24.0)	19.0 (12.0–27.5)	17.5 (15.0–20.0)	1.000	21.0 (16.8–22.5)
TAPSE	16.0 (15.0–20.3)	18.0 (15.0–21.0)	16.0 (15.3–19.8)	0.826	15.0 (13.3–19.5)
Right heart catheterization^a^					
RA pressure (mmHg)	5.5 (3.0–10.5)	7.5 (3.0–13.5)	5.0 (2.5–7.5)	0.392	5.0 (1.8–9.5)
Systolic PAP (mmHg)	44.5 (38.8–49.8)	38.0 (23.3–59.8)	33.5 (25.3–44.0)	0.165	36.0 (30.0–44.5)
Diastolic PAP (mmHg)	25.0 (20.8–29.5)	20.0 (11.0–35.3)	18.0 (13.3–22.0)	0.042	15.5 (12.0–21.5)
Mean PAP (mmHg)	32.5 (27.8–36.0)	28.0 (15.0–45.8)	25.0 (18.3–28.0)	0.238	23.5 (21.8–30.8)
PCWP (mmHg)	13.5 (10.5–29.0)	19.5 (7.5–38.8)	13.0 (11.3–16.3)	0.831	14.0 (10.5–22.5)
Cardiac output (L/min)	3.89 (3.48–5.25)	3.7 (3.3–6.8)	4.1 (3.8–4.7)	0.670	4.6 (3.6–5.0)
HF therapy at baseline					
Loop diuretics	10 (100.0)	6 (100.0)	4 (100.0)	‐	19 (95.0)
Beta‐blocker	9 (90.0)	5 (83.3)	4 (100.0)	1.000	17 (85.0)
Vasodilators	7 (70.0)	4 (66.7)	3 (75.0)	1.000	13 (65.0)
MRA	7 (70.0)	4 (66.7)	3 (75.0)	1.000	18 (90.0)
Anticoagulation	100 (100.0)	6 (100.0)	4 (100.0)	‐	17 (85.0)
ICD therapy	100 (100.0)	6 (100.0)	4 (100.0)	‐	19 (95.0)
CRT‐D	7 (70.0)	4 (66.7)	3 (75.0)	1.000	12 (60.0)

Abbreviations: BMI, body mass index; BP, blood pressure; CABG, coronary artery bypass graft; COPD, chronic obstructive pulmonary disease; CRT, cardiac resynchronization therapy; CVA, cerebrovascular accident; e‐GFR, estimated glomerular filtration rate; ICD, implantable cardioverter defibrillator; MRA, mineralocorticoid receptor antagonist; PAP, pulmonary artery pressure; PCWP, pulmonary capillary wedge pressure; RA, right atrial; TAPSE, tricuspid annular plane systolic excursion; TIA, transit ischemic attack.

^a^ Assess during CardioMEMS implantation for the CardioMEMS cohort or during LVAD screening for the Historical cohort.

### 3.1 Safety

There were no sensor failures, device‐related complications, or signal malfunctions at 1‐year of follow‐up.

### 3.2 Changes in pulmonary artery pressure

**Figure 1:**
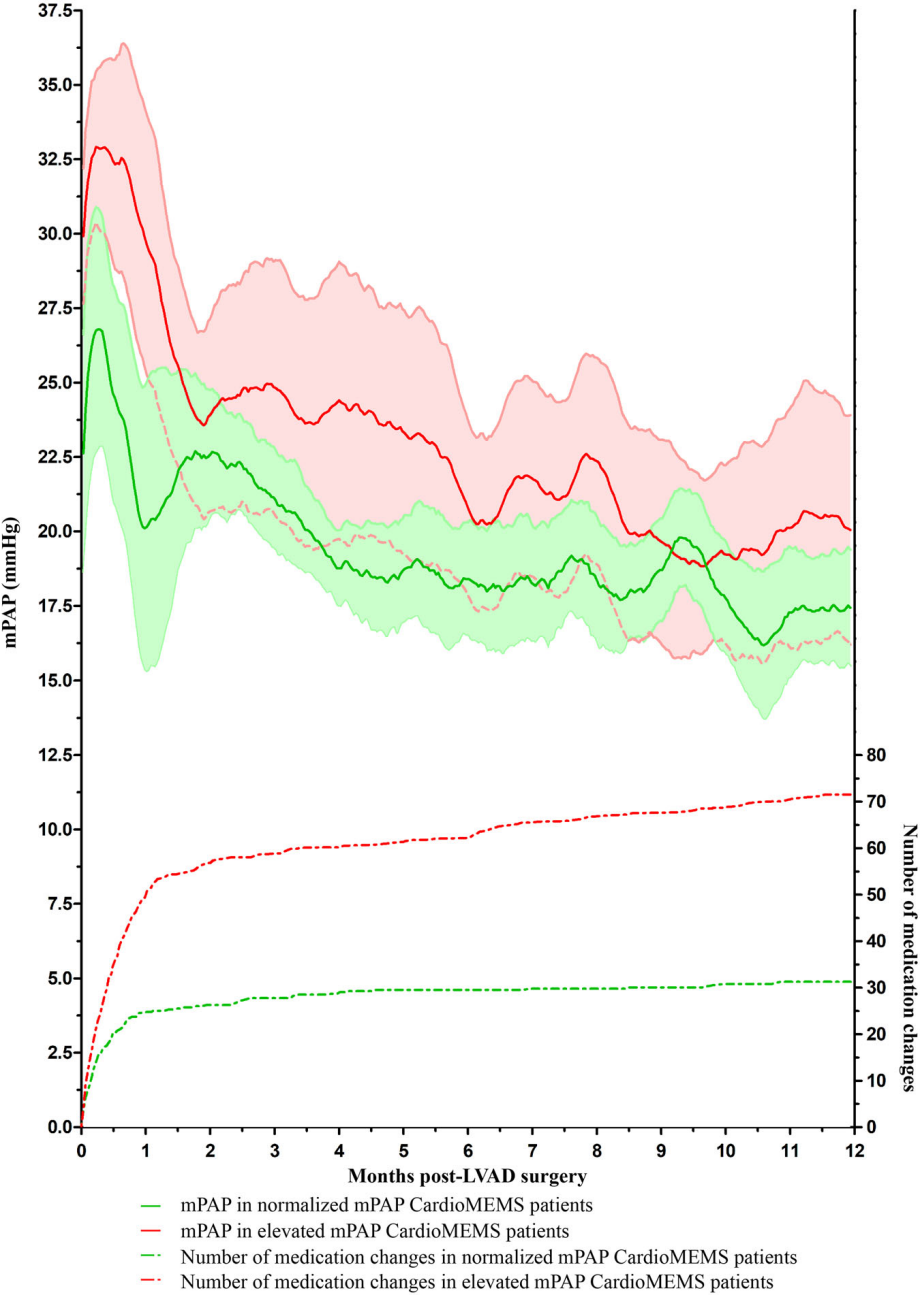
mean PAP and number of medication changes during the first year post‐LVAD surgery according to mPAP status pre‐LVAD surgery. LVAD, left ventricular assists device; mPAP, mean pulmonary artery pressure; PAP, pulmonary artery pressure

The mPAP over time and the number of medication changes in both CardioMEMS groups are shown in Figure 1. During this period, the average number of medication changes in the normal mPAP CardioMEMS patients was 33 (25–36), and 61 (45–104) in the elevated mPAP group (*p* = .114).

### 3.3 Clinical endpoints at 1‐year follow‐up

**Figure 2:**
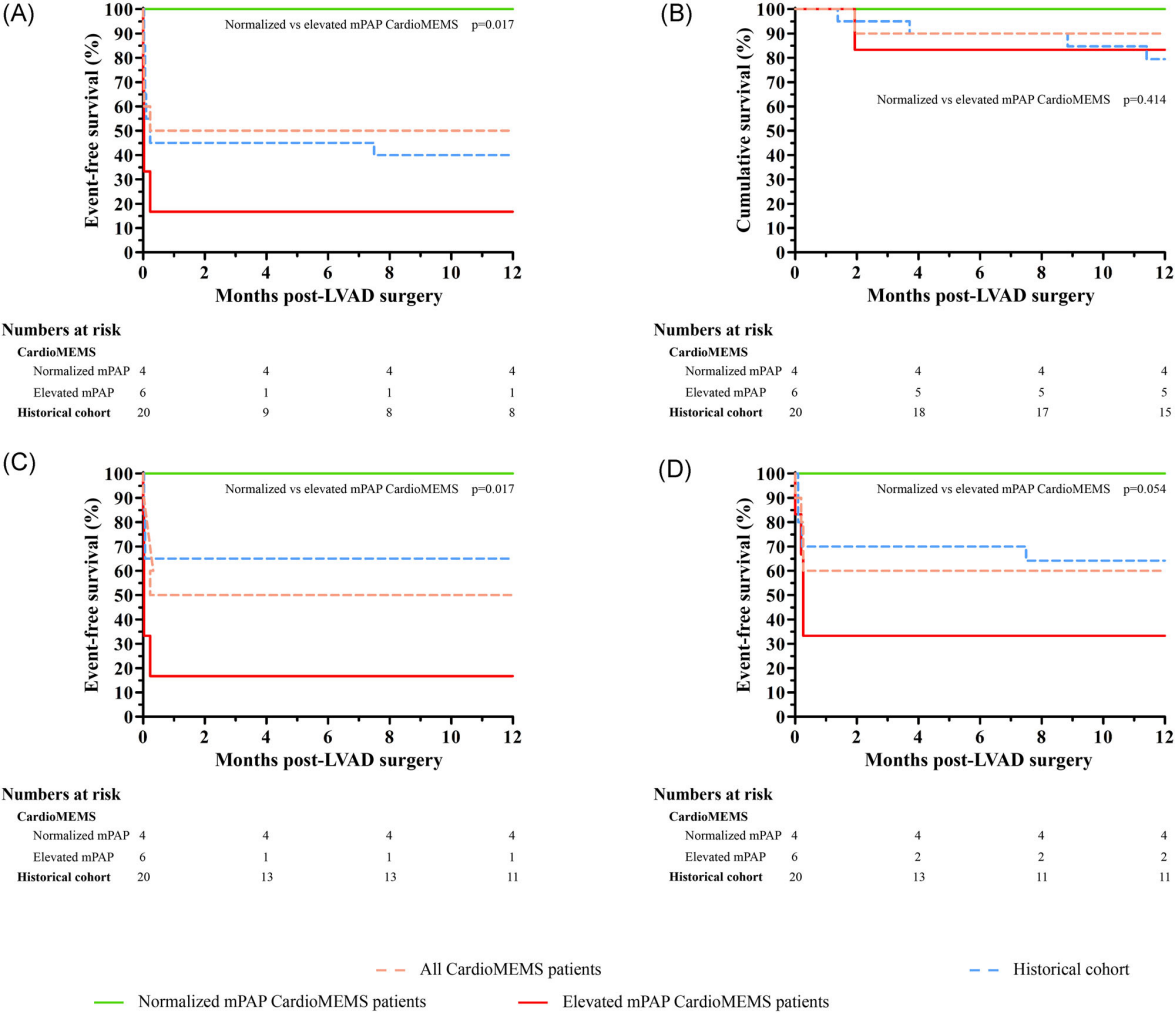
Event‐free survival of (A) the combined endpoint (all‐cause mortality, acute kidney injury and/or renal replacement therapy, and right ventricular failure), (B) survival, (C) acute kidney injury and/or renal replacement therapy, and (D) right ventricular failure in CardioMEMS patients and historical cohort. LVAD, left ventricular assists device

The combined endpoint (consisted of all‐cause mortality, AKI and/or the need for RRT, and/or RV‐failure) occurred in 50.0% of the total CardioMEMS group, of which 83.3% in elevated mPAP group and 0.0% in normalized mPAP group, with a relative risk difference of 83.3%, *p* = .017 (Figure 2A). The combined endpoint occurred in 60.0% of the historical cohort.

The survival was 90.0% in the total CardioMEMS groups, of which 100% of patients survived in the normalized mPAP group compared to 83.3% in the elevated mPAP groups, *p* = .41 (Figure 2B). AKI/RRT occurred in 50.0% of the total CardioMEMS groups, of which 0% in the normalized mPAP group and 83.3% in the elevated mPAP groups (*p* = .017, Figure 2C). RV failure occurred in 60.0% in the total CardioMEMS groups, of which 0% in the normalized mPAP group and 66.7% in the elevated mPAP groups (*p* = .054, Figure 2D). In the historical cohort, survival was 79.4%, AKI/RRT occurred in 35%, and RV‐failure in 35.8%.

### 3.4 Hospitalization‐free survival at 1‐year follow‐up

**Figure 3:**
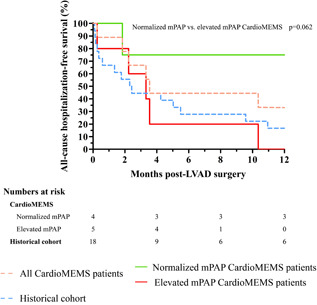
All‐cause hospitalization‐free survival in CardioMEMS patients and historical cohort. LVAD, left ventricular assists device; mPAP, mean pulmonary artery pressure

The all‐cause hospitalization‐free survival (at 1 year) of the cohorts is shown in Figure 3. The number of HF hospitalizations was significantly lower in the CardioMEMS patients who went into surgery with normal mPAP (0.0 [0.0–0.8]) compared to patients with an elevated mPAP (2.0 [2.0–3.0], *p* = .022).

### 3.5 Quality of life and functional performance

**Figure 4:**
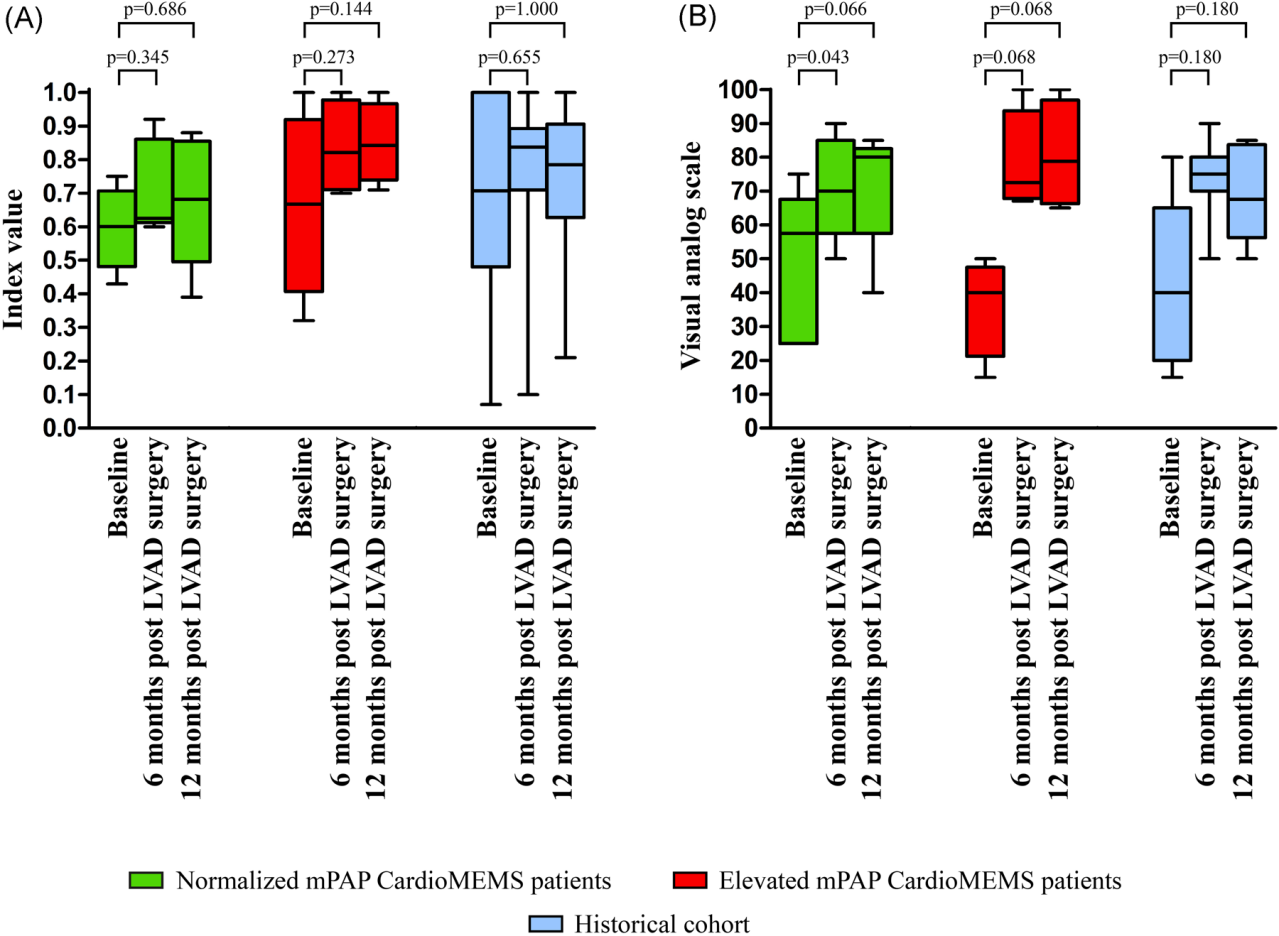
Changes in quality of life between baseline and 6 and 12 months post‐LVAD surgery (A) Index value (EQ‐5D‐5L) (B) Self‐reported quality of life (EQ‐5D‐5L) in CardioMEMS patients and historical cohort. Winker indicate the minimum and maximum values. LVAD, left ventricular assists device; mPAP, mean pulmonary artery pressure

CardioMEMS patients had a larger improvement in their quality of life, assessed by the EQ‐5D‐5L questionnaire (45.0 [25.0–61.3] to 80.0 [67.5–86.3], *p* = .011), compared to the historical cohort (40.0 [20.0–65.0] to 67.5 [56.3–83.8], *p* = .027). The changes in quality of life in the three patient cohorts are shown in Figure 4 separately.

**Figure 5:**
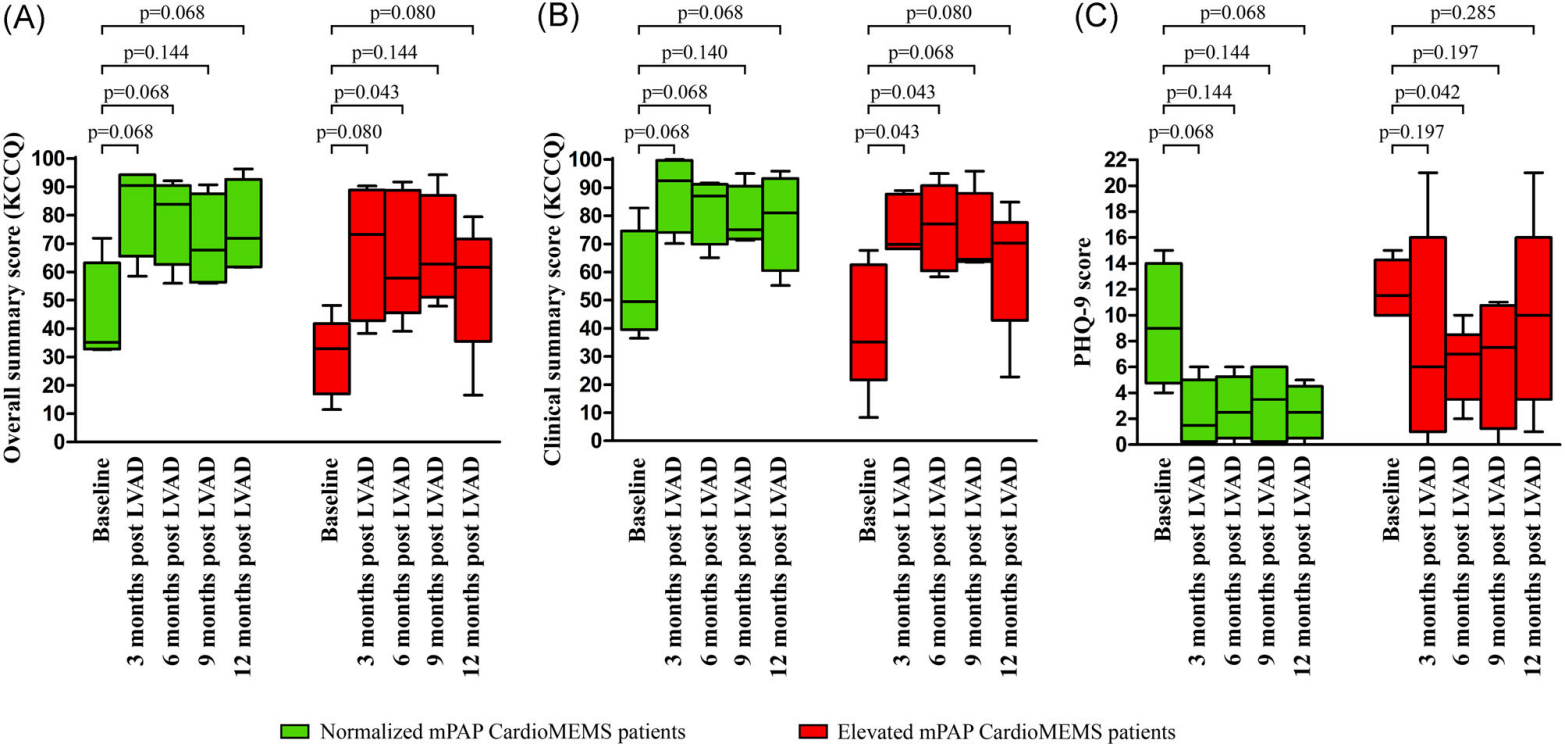
Changes in quality of life between baseline and 3, 6, 9, and 12 months post‐LVAD surgery (A) KCCQ overall summary score, (B) KCCQ clinical summary score, and (C) PHQ‐9 depression score. Winker indicate the minimum and maximum values. KCCQ, Kansas City cardiomyopathy questionnaire; LVAD, left ventricular assists device; mPAP, mean pulmonary artery pressure; PHQ‐9, patient health questionnaire‐9

In all CardioMEMS patients, the QoL assessed by the Kansas City Cardiomyopathy questionnaire overall summary score (34.9 [26.8–41.7] to 62.0 [58.1–80.6], *p* = .011) and clinical summary score (42.7 [32.3–62.6] to 70.3 [59.1–85.2], *p* = .015) as well as the PHQ‐9 depression score (11.0 [9.3–14.3] to 5.0 [1.5–10.5], *p* = .051) improved significantly over time. However, the improvement in the patients with a normalized mPAP before LVAD surgery was bigger compared to the patients with an elevated mPAP (Figure 5). The 6 min walking distance improved significantly from baseline up to 12 months post‐LVAD surgery in all CardioMEMS patients (264.0 [216.0–299.5) to 613.0 [476.3–695.8], *p* = .012). The improvement in walking distance was larger in normal mPAP patients (275.5 [149.4–377.0] to 690.0 [578–724.8], *p* = .068) compared to elevated mPAP patients (245.5 [216–299.5] to 493.5 [453.8–651.0], *p* = .068, Figure S1).

## 4 COMMENT

The results from this pilot concept study demonstrate the safety and feasibility of combining PAP monitoring and HM3 management as a hybrid construction inpatient management before and after surgery as well as on the out‐patient clinic. Most interestingly, the hemodynamic data identifies, with a clinically intuitive parameter, such as PAP, a high‐risk group of patients with a worse outcome of LVAD surgery and more HF hospitalizations during follow‐up. Likewise, patients with normal and stable pressures identify a low‐risk group with good prognosis and low hospitalization rates. This is clinically plausible but can especially help to guide LVAD management at the ICU phase and focus or intensify hospital recourses in high‐risk patients in an early stage as well as provide additional tools to anticipate with early and timely support of the RV. Additional hemodynamic data in this complex patient group would help to differentiate low and high risk patients and reduce the workload of larger growing LVAD programs and improve patient outcome.

### 4.1 Improving the long‐term survival

Due to technological improvements and advances in LVAD management, the overall survival of continuous‐flow LVAD patients has improved significantly over the last years, with 1‐year survival rates estimated between 75% and 85%.^2^, ^12^, ^13^, ^14^ However, LVAD‐related complications, such as severe RV‐failure, renal failure, and major bleedings events still affect the overall outcome of LVAD patients.^2^ Effective strategies to identify and optimize patients at high risk for LVAD complications are urgently needed. While our results from a small pilot concept study can only be used as hypothesis‐generating, which we emphasize here, we do also acknowledge that using hemodynamic feedback is clinically intuitive and clearly identifies a risk marker for adverse outcome in these patients. Especially patients with normal PAP or good response in PAP before surgery have a good outcome, most likely due to the true decongestion of the RV and venous renal pressure, which is important before surgery.

Adding daily PAP feedback in outpatient clinics, on top of the regular physical, laboratory, and regular echocardiographic investigations, will also reveal new insight into the course of treatment of this complex patient group in larger pivotal studies.

Some reports note that one can use a Swan Ganz catheter preoperatively and postoperatively in the management of LVAD patients.^15^ However, this strategy is severely limited by the invasive nature of the Swan‐Ganz measurements, with its own related complications and is only limited to use of several days with the risk of bleeding and infection proceeding towards LVAD surgery, with artificial material which is easily infected. The CardioMEMS device, with venous entry, is low risk, safe and easy to use at any moment, and stays permanently in the vessel, which overcomes the limitations of Swan Ganz and allows for easy repetition of hemodynamic optimization daily at the ICU but also for years of out‐patient management and at home.

### 4.2 Reducing readmissions

Although LVAD therapy improves the overall survival of end‐stage HF patients, even after LVAD surgery, the hospitalization rates of these patients remain enormously high. Approximately 30% of the LVAD recipients are readmitted within 30 days after their initial discharge,^16^, ^17^ and even more than 60%–80% is at least once readmitted 1 year after their initial discharge.^18^ On average, LVAD patients are admitted twice during the first year on LVAD support.^6^, ^19^ These frequent hospitalizations have a significant negative impact on the quality of life of these patients and places a large burden on hospital resources.

Further opportunities also lay in the long term assessment of pulmonary hypertension. Many patients with irreversible pulmonary hypertension (not responsive to vasodilators) receive LVAD therapy as destination therapy. Still, several reports^20^ also show that with several months to up to a year of continuous LV unloading, some patients do reverse PAP and become eligible for heart transplantation. Additionally, patients on the waiting list for heart transplantation with borderline pulmonary hypertension or rising PAP need to be studied with Swan Ganz measurements every 6 months.^21^ With average waiting times fort heart transplantation in Western Europe going to 2–3 years, CardioMEMS and continuous PAP monitoring van have a major role in reducing HF hospitalizations and repetitive invasive procedures in these patients.

During LVAD support, reports also note that decoupling between the diastolic PAP and pulmonary capillary wedge pressure (PCWP) can occur, resulting in elevated diastolic PAP with normal PCWP.^22^ In patients with decoupling, hemodynamic guided therapy using the CardioMEMS, which does not provide information on the PCWP, will need more caution as this might lead to excessive up‐titration of HF therapy. Additionally, the decoupling of PAP and PCWP has been associated with a higher chance of mortality and/or HF hospitalizations, especially if the decoupling increases during hemodynamic optimization of LVAD patients.^22^, ^23^

### 4.3 Improving the quality of life and functional performance

With the increasing survival of patients receiving LVAD therapy and the significant impact of LVAD‐related complications and changes in lifestyle, quality of life and functional performance become increasingly important. Several studies reported an initial increase in quality of life and functional performance only during the first 6 months post‐LVAD surgery,^24^, ^25^ but no further improvement after the first 6 months.^26^

## 5 LIMITATIONS

We acknowledge that patients with severe right ventricular dysfunction were excluded as well as the small sample size of our pilot study, which is hypothesis‐generating, to test the safety and feasibility of a novel concept to use a PAP sensor with HM3 continuous data as a hybrid construction for the first time in a prospective study. Intuitively, with such a clinical parameter, it makes sense to add the hemodynamic feedback to the static pump management, as it adds information to individualized management even at home. The current pilot study confirms the safety and feasibility, but the additive value will need to be tested in large scale pivotal studies.

## 6 CONCLUSION

Remote hemodynamic monitoring in LVAD patients is safe and feasible and could be used to provide physicians involved in LVAD care at different stages with incremental information that can be used to identify patients at elevated risk of complications as well as optimize patient management remotely during the out‐patient phase. The CardioMEMS sensor provides a clinically interpretable risk stratifier for adverse outcome. Larger pivotal studies are warranted to test the hypothesis generated from this pilot study on remote hemodynamic monitoring with CardioMEMS in the LVAD population.

## CONFLICT OF INTERESTS

Jasper J. Brugts received speaker fee from Abbott. Nicolas M. van Mieghem received speaker fee from Abbott, Boston Scientific, Edwards Lifesciences, Medtronic, Daiichi Sankyo, PulseCath BV, Teleflex. Jesse F. Veenis, Sumant P. Radhoe, Olivier C. Manintveld, Jos A. Bekkers, and Kadir Caliskan declared no conflict of interest on this topic.

## Supporting information

Supporting information.
