# Supplementary material for: Safety and feasibility of hemodynamic pulmonary artery pressure monitoring using the CardioMEMS device in LVAD management
Source: J Card Surg. 2021 Jun 22;36(9):3271–80. doi: 10.1111/jocs.15767 (PMC8453715; doi:10.1111/jocs.15767)
Supplement: Supplementary file 1 — Supporting information. [file JOCS-36-3271-s001.docx]

**Safety and feasibility of a hybrid construction of hemodynamic guidance by pulmonary artery pressure monitoring and LVAD management:**

**Main findings of the proof of concept HEMO-VAD study**

Jesse F. Veenis, Sumant P. Radhoe, Nicolas M. van Mieghem, et al.

**Table of content**:

**Figure S1.** Changes in 6-minute walking distance at baseline, discharge and 3, 6, 9 and 12 months of follow-up in CardioMEMS patients

**Figure S1.** Changes in 6-minute walking distance at baseline, discharge and 3, 6, 9 and 12 months of follow-up in CardioMEMS patients

**
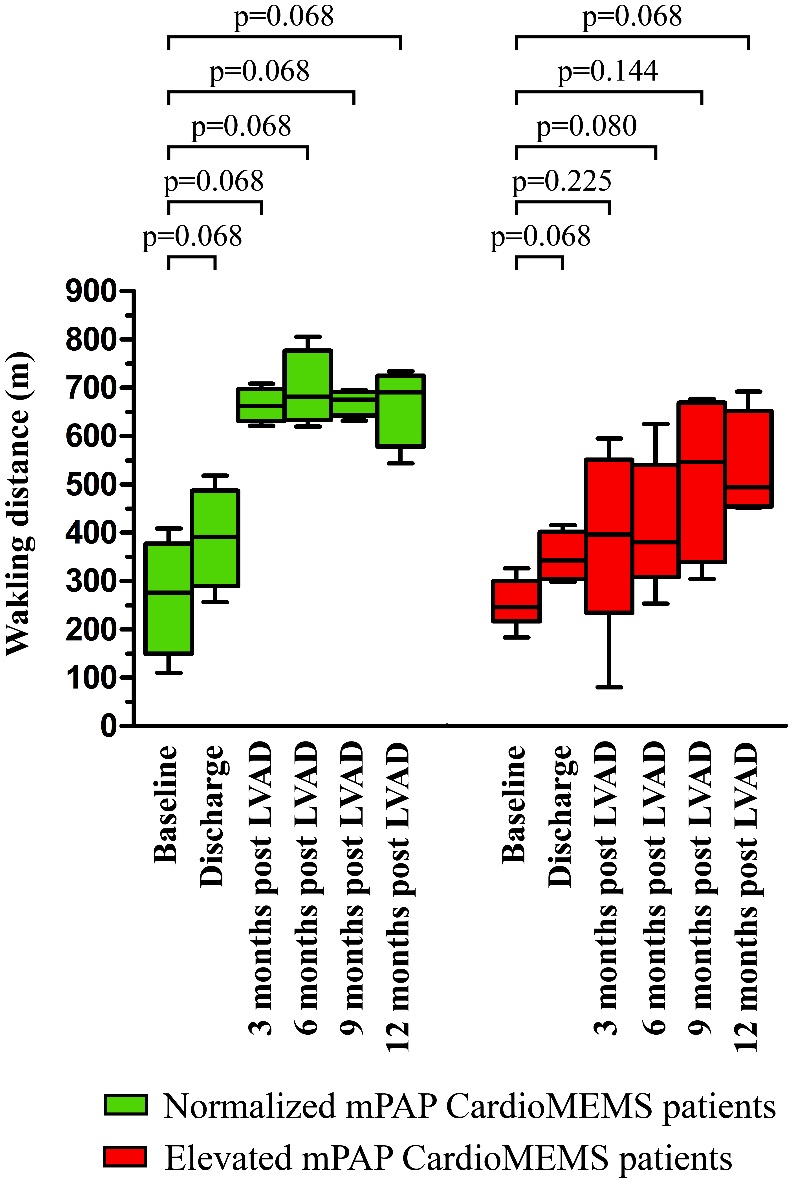
**

*Winker indicate the minimum and maximum values*
